# Multi-Stage Harmonization for Robust AI across Breast MR Databases

**DOI:** 10.3390/cancers13194809

**Published:** 2021-09-26

**Authors:** Heather M. Whitney, Hui Li, Yu Ji, Peifang Liu, Maryellen L. Giger

**Affiliations:** 1 Department of Radiology, The University of Chicago, Chicago, IL 60637, USA; huili@uchicago.edu (H.L.); jiyu@tjmuch.com (Y.J.); 2 Department of Physics, Wheaton College, Wheaton, IL 60187, USA; 3 Tianjin Medical University Cancer Institute and Hospital, Tianjin 300060, China; liupeifang@tjmuch.com

**Keywords:** computer-aided diagnosis, radiomics, breast cancer, harmonization, magnetic resonance imaging, machine learning

## Abstract

**Simple Summary:**

Batch harmonization of radiomic features extracted from magnetic resonance images of breast lesions from two databases was applied to an artificial intelligence/machine learning classification workflow. Training and independent test sets from the two databases, as well as the combination of them, were used in pre-harmonization and post-harmonization forms to investigate the generalizability of performance in the task of distinguishing between malignant and benign lesions. Most training and independent test scenarios were statistically equivalent, demonstrating that batch harmonization with feature selection harmonization can potentially develop generalizable classification models.

**Abstract:**

Radiomic features extracted from medical images may demonstrate a batch effect when cases come from different sources. We investigated classification performance using training and independent test sets drawn from two sources using both pre-harmonization and post-harmonization features. In this retrospective study, a database of thirty-two radiomic features, extracted from DCE-MR images of breast lesions after fuzzy c-means segmentation, was collected. There were 944 unique lesions in Database A (208 benign lesions, 736 cancers) and 1986 unique lesions in Database B (481 benign lesions, 1505 cancers). The lesions from each database were divided by year of image acquisition into training and independent test sets, separately by database and in combination. ComBat batch harmonization was conducted on the combined training set to minimize the batch effect on eligible features by database. The empirical Bayes estimates from the feature harmonization were applied to the eligible features of the combined independent test set. The training sets (A, B, and combined) were then used in training linear discriminant analysis classifiers after stepwise feature selection. The classifiers were then run on the A, B, and combined independent test sets. Classification performance was compared using pre-harmonization features to post-harmonization features, including their corresponding feature selection, evaluated using the area under the receiver operating characteristic curve (AUC) as the figure of merit. Four out of five training and independent test scenarios demonstrated statistically equivalent classification performance when compared pre- and post-harmonization. These results demonstrate that translation of machine learning techniques with batch data harmonization can potentially yield generalizable models that maintain classification performance.

## 1. Introduction

For a given medical imaging protocol, differences in the resulting medical images and the values extracted from them can arise when they are collected in different contexts. For example, different manufacturers can implement a protocol that is nominally the same but then additionally apply proprietary algorithms which may affect the exported data, or some factors in the imaging protocol may be systematically different between two groups, such as magnetic resonance imaging at different clinical field strengths. In general, medical imaging conducted in different contexts may demonstrate several types of differences, deriving from factors such as patient biology, screening protocols, and imaging protocols [1]. Many artificial intelligence/computer-aided diagnosis (AI/CADx) models for diagnosis and prognosis of disease make use of medical images that are acquired within a single institution. This can potentially reduce some differences in factors, however, there is substantial interest in combining datasets to form potentially more generalizable models through the use of images from multiple institutions.

Harmonization is an area of investigation in AI that seeks the compatibility of data acquired from different contexts or sources. The concept of harmonization can have several different levels of application in medical imaging, such as at image acquisition, in which protocols are controlled to be implemented the same way in different contexts, or in the post-processing of acquired images to normalize them between two sources of data.

**Figure 1:**
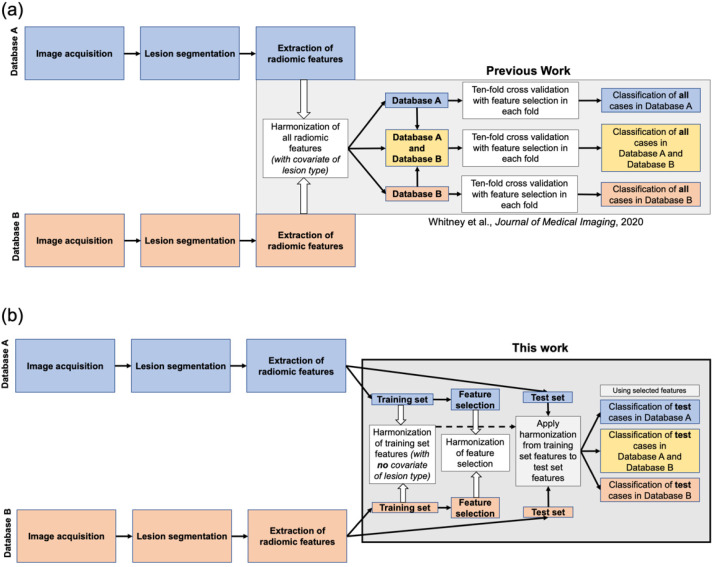
Illustration of two stages in an AI/CADx medical imaging workflow where harmonization can be applied. (**a**) Previous work investigated the impacts of batch harmonization in a single stage of all features in the two databases with covariates in the context of cross-validation (Whitney et al., *Journal of Medical Imaging* 2020 [5]). (**b**) This present work investigates both batch harmonization of features and harmonization of feature selection in a training and independent test framework (i.e., harmonization is conducted on the features of the lesions in the training set and separately applied to the features of the lesions in the test set), with no covariates.

One level of combining datasets from different sources involves the batch harmonization of extracted features. Batch harmonization methods seek to reconcile what are termed batch effects in the field of genomics, the observation that data drawn from otherwise identical samples measured at different times can show some differences in values [2]. Harmonization can be applied to sets of radiomic features with the underlying assumption that the acquisition of features from images acquired with nominally similar protocols but from different sources may demonstrate these batch effects. In the context of AI/CADx, different databases can be considered as different batches. The harmonization method ComBat [2] has previously been used in a single stage on all cases in each database of radiomic features extracted from full-field digital mammography images [3], positron emission tomography images of breast cancer [4], dynamic contrast-enhanced magnetic resonance (DCE-MR) imaging of breast cancer [5] (Figure 1a), computed tomography images for lung cancer and phantom imaging [6,7], MR imaging of soft tissue sarcomas [8], measurements of diffusion tensor imaging [9] and cortical thickness [10] in the brain, and FLAIR and T1-weighted imaging of the brain and T2-weighted imaging of the prostate [11]. The application of ComBat harmonization to radiomic features of all cases in each database allows for the use of covariates, such that the characteristics of the batches, for example lesion type, are retained. These works and others have demonstrated that ComBat can potentially be useful when the goal is to harmonize, in one stage, entire datasets resulting from combined batches. A recent work summarizes the current status of investigations into harmonization of radiomic features [12].

A related potential application for batch harmonization is its application to an AI/CADx workflow involving training classifiers on a training set and applying them to a completely separate, independent test set. In such a scenario, batch harmonization would be conducted first on the training set alone, with the parameters from the harmonization subsequently applied to the test set, i.e., the data in the test set do not influence the harmonization parameters. Applying harmonization in this way can have potential impacts not relevant when batch harmonization is applied to all cases in each database. For example, batch harmonization may affect feature selection, i.e., the features selected for combined sets of features from different batches may be different by using pre-harmonization features compared with those selected by using post-harmonization features. This may in turn affect classification of cases in the independent test set. Thus, the use of batch harmonization on a training set and then application of parameters to an independent test set requires an additional stage of feature selection harmonization, i.e., the features used in the test set must be the same as those selected on the training set (Figure 1b). Additionally, because theoretically such a workflow would use unlabeled test cases, covariates (such as, which cases are malignant or benign) cannot be used.

The purpose of this study was to assess the performance of AI/CADx of breast cancer in lesions imaged with DCE-MR on two databases using extracted human-engineered radiomic features, batch harmonization, feature selection harmonization, and classification. In this study, the two databases were considered to be the two batches for the purposes of batch harmonization. First, we investigated the effects of batch harmonization of features in the training set that combined the two batches (i.e., the two training datasets) and the application of the associated batch harmonization parameters to the features in the independent test sets. Next, we conducted feature selection and classification using the training and independent test set paradigms on the two databases separately and on combined versions of them, in both their pre-harmonization and post-harmonization forms. We hypothesized that an independent training/test AI/CADx lesion classification pipeline that both harmonizes features by databases and allows feature selection to change after batch harmonization (that is, feature selection harmonization) will result in a predictive model that is generalizable across the two databases, i.e., multi-stage harmonization maintains classification performance. 

## 2. Materials and Methods

### 2.1. Database

The study was performed retrospectively under IRB/HIPAA protocol, which waived requirement of informed consent. DCE-MR images of breast lesions from females were collected from two medical centers. These databases were termed Database A and Database B in this study. Lesions in Database A had been imaged during the period of 2005–2017 while the lesions imaged in Database B had been imaged during the period of 2015–2017. The lesions had been imaged using T1-weighted gradient spoiled sequences in use at the medical centers. Most lesions in Database A had been imaged using Philips Intera scanners, except for three benign lesions which had been imaged using GE scanners, and five and three cancerous lesions which had been imaged using GE and Siemens scanners, respectively. All lesions in Database B had been imaged using GE Discovery 750 scanners. Images from Database A had been acquired in the axial plane and images from Database B were acquired in the sagittal plane. The details of the imaging protocols are available elsewhere [5,13,14,15]. Information regarding subtypes of benign lesions and cancers had been collected from pathology and imaging reports.

**Figure 2:**
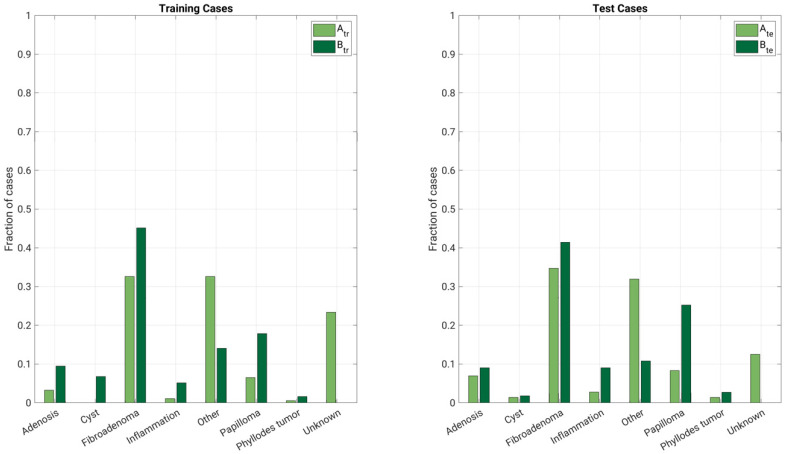
Description of the dataset: fraction of types of benign lesions (one lesion per case) by database (A: Database A; B: Database B; tr: training; te: test).

**Figure 3:**
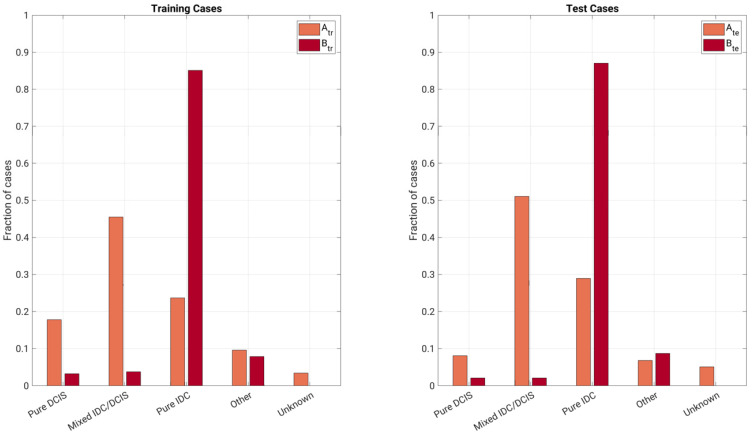
Description of the dataset: fraction of types of cancers (one lesion per case) by database (A: Database A; B: Database B; tr: training; te: test; DCIS: ductal carcinoma in situ; IDC: invasive ductal carcinoma).

**Figure 4:**
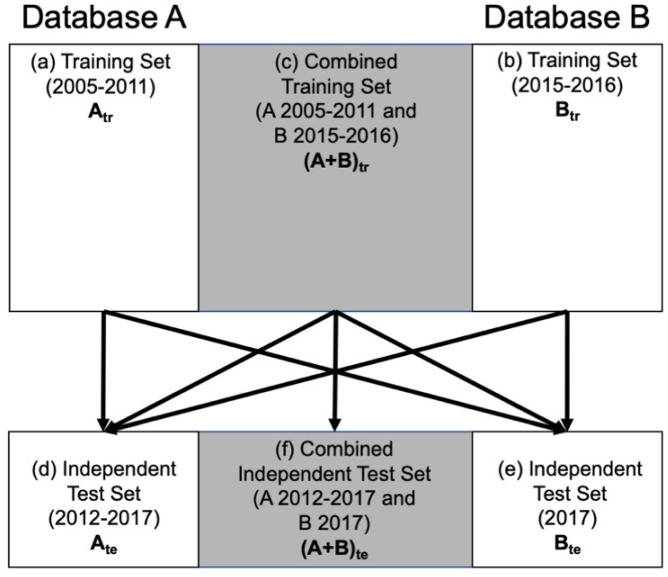
Framework for independent training and test between each set. Each arrow originates in a training set and terminates in an independent test set. Years of image acquisition are indicated in parentheses.

The training and test sets of Database A and Database B were determined based solely on year of image acquisition (Table 1, Figure 2 and Figure 3). These training and independent test sets from each database were also combined, respectively, yielding a combined training set and a combined test set. Thus, there were three separate training sets: (a) lesions in Database A imaged between 2005 and 2011, (b) lesions in Database B between 2015 and 2016, and (c) the combination of these. Similarly, there were three independent test sets: (d) lesions in Database A imaged between 2012 and 2017, (e) lesions in Database B imaged in 2017, and (f) the combination of these (Figure 4). This framework was chosen because of the different types of potential uses of a harmonized training set. 

### 2.2. Lesion Segmentation and Feature Extraction 

Lesions had previously been segmented on the MRIs using a computerized fuzzy C-means method [16]. In addition, thirty-two radiomic features had already been automatically extracted subsequently after lesion segmentation. The features were made up of five phenotypic feature categories: size, shape, morphology, enhancement texture, and kinetic curve assessment [17,18,19]. These techniques and performance levels had previously been reported.

### 2.3. Harmonization

The batch harmonization method used in this work, Combat [3], models feature data across two batches as (Equation (1)):

(1) $$Y_{ijg}=\alpha_{g}+X\beta_{g}+\gamma_{ig}+\delta_{ig}\varepsilon_{ijg}$$

where $\alpha_{g}$ is the average value for feature *g*, X is a design matrix for the covariates of interest, $\beta_{g}$ is the vector of regression coefficients corresponding to each covariate, $\gamma_{ig}$ is the additive effect of group *i* on feature *g*, $\delta_{ig}$ is a multiplicative group effect, and $\varepsilon_{ijg}$ is an error term for each sample *j*. The data are standardized according to (Equation (2)):

(2) $$Z_{ijg}=\frac{Y_{ijg}-{\hat{\alpha }}_{g}-X\beta_{g}}{{\hat{\sigma }}_{g}}$$

where $\hat{\alpha }$ and $\hat{\beta }$ are estimators of α and β, respectively.

The variables $\gamma_{ig}$ and $\delta_{ig}$ are then estimated using empirical Bayes estimates. Then, each feature is transformed using the expression (Equation (3)):

(3) $$Y_{ijg}^{Combat}{}_{}=\frac{{\hat{\sigma }}_{g}}{{\hat{\delta }}_{ig}^{*}}\left(Z_{ijg}-{\hat{\gamma }}_{ig}^{*}\right)+{\hat{\alpha }}_{g}+X{\hat{\beta }}_{g}$$

The ComBat harmonization method was applied to radiomic features extracted from lesions in the training set made of the combined databases, i.e., (A + B)_tr_. In that process, the empirical Bayes estimates needed to shrink the batch effect parameter estimates of mean and variance were identified. Subsequently, those parametric Bayes estimates (${\hat{\gamma }}_{ig}^{*}$ and ${\hat{\delta }}_{ig}^{*}$) and the factors ${\hat{\alpha }}_{g}$ and ${\hat{\sigma }}_{g}$ from the training set were then used to harmonize the eligible features in the independent test set made of the combined database, i.e., (A + B)_te_. 

Batch harmonization determines harmonization factors from all features used in harmonization, so ComBat harmonization was conducted by feature category on collections of features deemed eligible for harmonization, namely, morphology, texture, and most kinetic curve features. That is, harmonization was conducted for morphology features, then for texture features, and then for most kinetic curve features. Kinetic curve features with semi-categorical variables (washout rate and curve shape index) did not undergo harmonization, nor did the kinetic curve feature of volume of most enhancing voxels (Table 2). It is important to note that since the predictive modeling process would theoretically involve unlabeled test cases if used in a clinical workflow, no covariates were used in the harmonization.

The complete compilation of the full feature sets included both harmonized features and the features that did not undergo harmonization, i.e., those that remained as is in the feature sets.

After harmonization, the training set comprised of both batches, (A + B)_tr_, was separated into its pre-harmonization and post-harmonization separate database forms (A_tr_ and B_tr_). After harmonization parameters from (A + B)_tr_ were applied to (A + B)_te_, (A + B)_te_ was separated into its pre-harmonization and post-harmonization separate database forms (A_te_ and B_te_).

As an intermediate step, the visualization of feature harmonization on the training and independent test sets was investigated using t-distributed stochastic neighbor embedding (tSNE) [20,21].

### 2.4. Feature Selection

To enable classifier training, stepwise feature selection was conducted on the three training sets in their pre-harmonization and post-harmonization forms, to yield six sets of selected features, i.e., three with pre-harmonization selected features for A_tr_, B_tr_, and (A + B)_tr_, and three with post-harmonization selected features for A_tr_, B_tr_, and (A + B)_tr_.

### 2.5. Lesion Classification

Linear discriminant analysis (LDA) was used as the classifier to yield an estimate of the posterior probability of malignancy (PM) for each lesion. The determination of the LDA weights was computed using the training sets for ultimate use in the independent evaluation on the test sets (Figure 4). That is, six sets of LDA weights were calculated corresponding to the three pre-harmonization training sets of A_tr_, B_tr_, and (A + B)_tr_, and to the three post-harmonization training sets of A_tr_, B_tr_, and (A + B)_tr_.

The area under the receiver operating characteristic (ROC) curve (AUC) [22] for the task of classification of lesions as benign or malignant using the proper binormal model [23] served as the figure of merit with the PM as the input. Note that ROC analysis was conducted on each of the training/test scenarios in Figure 4.

### 2.6. Classification Performance Comparison on the Test Sets

Classification performance was compared using superiority testing for each of the combinations of training and testing shown in Figure 4 under both pre-harmonization and post-harmonization conditions. The Bonferroni correction of *p*-value for multiple comparisons [24] was utilized for each of the test sets, i.e., A_te_ and B_te_. Thus, in these cases the difference in AUC was deemed to be statistically significant if *p* < 0.025 (i.e., *p* < 0.05/2 comparisons). *p*-value correction was not necessary for the independent test set made up of lesions from both countries (A + B)_te_ since it was evaluated only once, so the difference in AUC was statistically significant if *p* < 0.05. Equivalence margins for conditions of similarity testing (equivalence and if necessary, non-inferiority) were identified *a posteriori* when a result failed to demonstrate significant difference [25], because the equivalence margin had not been identified for this classification task.

## 3. Results

### 3.1. Visualization of Feature Value Harmonization

**Figure 5:**
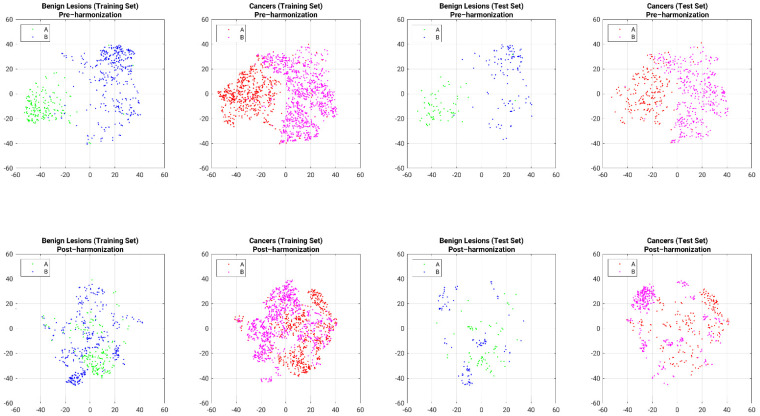
Feature value harmonization visualized through t-SNE presentations of pre-harmonization (top row) and post-harmonization (bottom row) by lesion type (benign lesion or cancer) and feature set (training or test set). Harmonization was conducted on the combination of benign lesions and cancers in the training set without covariate of lesion type and applied to lesions in the test set without covariate of lesion type. Results shown here are separated out by lesion type (cancer or benign) to aid in visualization only (A: Database A; B: Database B).

t-SNE figures demonstrate similar impact of harmonization of features in both the training and test sets, as seen by a reduction in the separation of the lesion features across institution, for both benign lesions and cancers, and in both the combined training and the combined test sets (Figure 5).

### 3.2. Feature Selection

**Figure 6:**
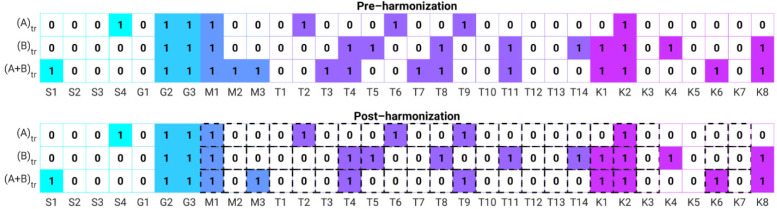
Feature selection by feature sets used in the study. Top: pre-harmonization features; bottom: post-harmonization features. Abbreviations in the figure correspond to the features listed in Table 2. Squares outlined with a dashed line indicate those for which harmonization was conducted.

Only when using the combined dataset under pre-harmonization and post-harmonization conditions did feature selection demonstrate some differences in the selected features (Figure 6). This contrasted with no change in selected features when using either Dataset A or Dataset B between the pre-harmonization and post-harmonization features. Notably, most of the features that exhibited a change in selection or non-selection were texture features. Texture features T3, T7, T8, and T11 were not selected post-harmonization but were selected pre-harmonization. Texture feature T9 was selected post-harmonization after having not been selected pre-harmonization.

### 3.3. Lesion Classification Performance and Comparison

As expected, within either Database A or Database B, classification performance (AUC: median, [95% CI]) was unchanged (within numerical calculation limits) when training and independent testing was performed on both pre-harmonization and post-harmonization feature sets (AUC_A_ = 0.872 [0.822, 0.915], AUC_B_ = 0.891 [0.853, 0.925]). 

**Figure 7:**
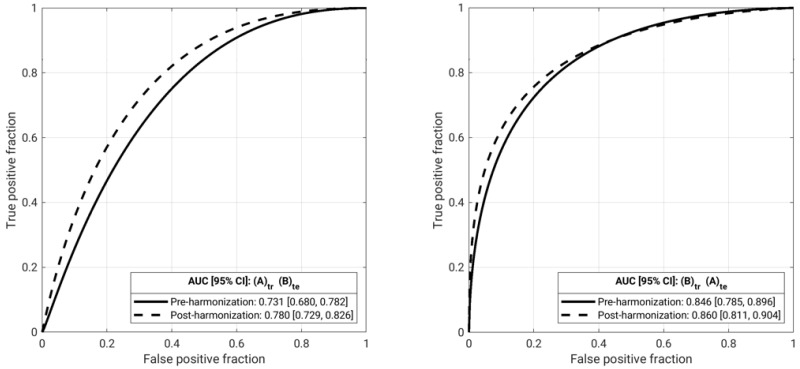
Classification performance results after batch feature harmonization and feature selection harmonization: Receiver operating characteristic (ROC) curves for training and testing between each database in the task of classification of lesions as malignant or benign, using a database-specific independent test set determined by date of image acquisition. Solid lines show the ROC curve when using pre-harmonization features, while dashed lines show the ROC curve when using post-harmonization features. AUC values are given in Table 3 (A: Database A; B: Database B).

Classification performance using features harmonized across the two databases demonstrated statistically significant difference when training of a classifier was conducted using lesions imaged in Database A and testing was conducted on lesions imaged in Database B, but not vice versa (Figure 7, Table 3). 

**Figure 8:**
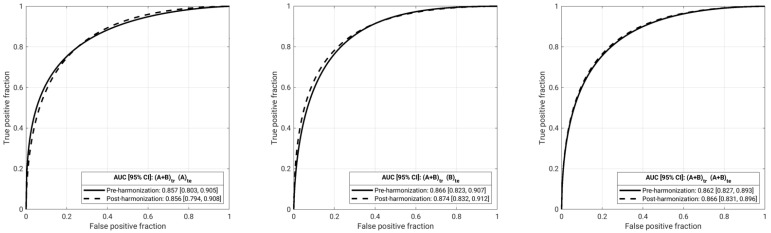
Classification performance results after batch feature harmonization and feature selection harmonization: Receiver operating characteristic (ROC) curves for the training and testing using a training set comprised of lesions from both databases. Solid lines show the ROC curve when using pre-harmonization features, while dashed lines show the ROC curve when using post-harmonization features. AUC values are given in Table 3 (A: Database A; B: Database B).

In contrast, there was no statistical evidence for difference in classification performance when training was conducted using combinations of features from both databases and independent testing was conducted using features from one dataset or the combination of them (i.e., (A + B)_te_), when comparing using pre-harmonization features to using post-harmonization features (Figure 8, Table 3). 

## 4. Discussion

The work presented here appears to be the first to demonstrate batch harmonization of radiomic features extracted from magnetic resonance images conducted in a training and independent test workflow and to investigate the impact of harmonization on classification performance in the context of both feature harmonization and feature selection harmonization. Statistically significant improvement in classification compared using pre- and post-harmonization features was observed in only one of the training and test combinations (A_tr_, B_te_). This may be due to the fact that feature harmonization overcomes the otherwise limiting number of features selected in A_tr._ The AI/CADx framework used in this study utilized both feature harmonization and feature selection harmonization, demonstrating that implementing these in the training and independent test framework maintains the generalizability of the predictive model. 

Batch harmonization via the ComBat method is being extensively utilized to harmonize entire databases of features in a single stage, as noted in the references mentioned above and in other recent works [26,27,28,29,30], but this restricts the application of that form of batch harmonization to cross-validation. In addition to the application of ComBat in its original form, variations are beginning to be investigated, such as M-Combat, which identifies one batch as the reference and harmonizes features in the second to the reference [31,32]. Our work described here is one of a few at this time that investigate the use of harmonization across batches (i.e., not to a reference set) in a completely separate training and test machine learning framework. Luo et al. used a separate training and test framework for various harmonization methods, including ComBat, in their study on microarray gene expression data [33]. The Matthews correlation coefficient (MCC) [34], a variation on the Pearson correlation coefficient, served as the figure of merit in the study, and a given batch harmonization method was determined to be better than no batch effect removal method if the difference in the MCC was greater than a pre-determined threshold. In that study, the ComBat method demonstrated utility in batch harmonization, but the conclusions were limited by the lack of statistical method to determine significance. Pszczolkowski et al. [35] used ComBat on separate training and test sets for a study that compared the use of radiomic features extracted from computed tomography images of the brain to using radiological signs (the blend sign, black hole sign, hypodensities, and island signs) and clinical factors to predict hematoma expansion and functional outcome with acute intracerebral hemorrhage. The authors demonstrated the effect of harmonization on feature distributions using t-SNE figures, resulting in a similar reduction in clustering across batches as presented here, but they did not report investigating the impact of ComBat in this way on classification using radiomic features alone, compared with not using ComBat. Radua et al. reported preparing software for the separate training and test framework [36] but their study did not implement it. Da-ano et al. [37] applied four variations of ComBat harmonization—the original version, M-Combat, B-Combat (a version that implements bootstrapping) and BM-Combat (a version that combines single reference and bootstrapping)—to radiomic features extracted from multiparametric magnetic resonance images (T2-weighted, apparent diffusion coefficient, DCE) and from positron emission tomography images of locally advanced cervical cancer for the prediction of local failure in 189 subjects. Their study compared the effect of harmonization on the entire set of features to harmonizing on a training set and applying it to a separate test set. Their results did not demonstrate statistically significant different performance in classification between these two scenarios, a finding which they believed to be favorable for the use of ComBat in a separate training and test framework. That study used only $\gamma_{ig}$ and $\delta_{ig}$ as the parameters determined by the training set and applied to the test set. The two other previous studies which describe using ComBat harmonization in completely separate training and test frameworks [33,35] do not specify which parameters they applied to the test set. In our study, we applied four parameters ($\gamma_{ig}$ and $\delta_{ig}$ as well as ${\hat{\alpha }}_{g}$ and ${\hat{\sigma }}_{g}$) to the test set. We also studied applying $\gamma_{ig}$ and $\delta_{ig}$ alone to the test set and found no statistical change in classification performance. This may be due to the relatively homogeneous nature of our database across the training and test sets, but it may be important to apply harmonization using the four parameters so that the determination of the harmonization parameters is completely separate from the test set, including those used for the standardization of test set features (as described in Equation (2)).

AI methods in MRI have been used to support decision making in breast cancer diagnosis and prognosis for several decades [38,39]. As the scope and sophistication of AI in medical imaging grows, some have noted that it would be beneficial to implement specific data science curriculum for radiology trainees [40,41]. Education in harmonization of data or feature selection could potentially be one element of such training, but at this time, the harmonization methods described here are in development and have not yet been approved for clinical use. 

There were several limitations to this study. For example, we did not investigate correlation with imaging protocol (such as field strength of imaging or image resolution) or patient biology. Note that our results are limited to the clinical task of classification of breast lesions as malignant or benign. Other studies have described statistically significant improvement in classification when using harmonization on entire databases and covariates (e.g., [4,5]), but our study did not include covariates due to its training and independent test design. Additionally, these results may be specific to the types of features used in this study, especially since some features were determined to not be eligible for harmonization. We elected to investigate these features for classification since they have been utilized in several studies for this particular classification question [42,43].

MRI is being investigated as a screening method for patients with increased lifetime risk of breast cancer, history of chest or mantle radiation therapy, history of breast cancer diagnosis and dense breasts, and previous diagnosis of breast cancer before age 50 [44]. However, our work did not study correlations across different screening protocols. Note that the primary goal of our method in eventual clinical use would be to provide decision-making support to radiologists. However, further validation and regulatory clearance are necessary prior to clinical implementation. In addition, our databases did not include male subjects. The incidence of breast cancer in males is approximately 1% in the United States [45]. Some case reports have indicated that MR imaging of breast cancers in males can provide decision-making support when initial imaging by mammography and ultrasound imaging is equivocal [46,47]. Future studies will investigate the inclusion of male breast cancer cases in training and/or test sets.

Our study did not investigate the performance of batch harmonization on lesion classification when more than two batches were used. It would be interesting to investigate the classification performance of a third batch of lesions in the testing capacity when they, as was true for our test sets, have no influence on the determination of the harmonization parameters, but they are also not present in the training sets. However, the choices of study design in this work may helpfully demonstrate classification performance in the context of limited scope of training and independent testing frameworks and provide a foundation for further studies.

Finally, while the classification performances in the test sets were maintained when compared pre- and post-harmonization, the ranking of individual cases within the test set could have changed (i.e., the posterior probability of malignancy of individual cases changed). This may have implications for the case-based repeatability of individual cases [48,49,50,51,52] and will be a topic of study in the future.

## 5. Conclusions

Classification performance using pre- and post-harmonization radiomic features extracted from DCE-MR images of the breast, in the task of classification of lesions as malignant or benign, has been demonstrated in computer-aided diagnosis using two training and independent test sets. The results suggest that both batch harmonization and its impact on feature selection may not provide statistically significant improvement in classification in training and independent test AI/ML workflows, but rather serve best to preserve classification performance for fixed feature selection. These findings demonstrate relevant considerations when designing methods of classification using machine learning for predictive models applied to differently acquired test sets, especially when attempting to combine datasets collected from two different sources.

## Figures and Tables

**Table 1:** Description of the dataset: number of lesions, age of subjects, and size of lesions, by database. The size of lesions is given as maximum linear size (extracted radiomic feature S4, see Table 2 below) (A: Database A; B: Database B; CI: confidence interval; min: minimum; max: maximum; tr: training; te: test).

	Training Set		Test Set	
Database	Benign	Cancer	Benign	Cancer
**A + B**	**(A + B)_tr_**		**(A + B)_te_**	
Number (% of set)	554 (24%)	1726 (76%)	183 (22%)	660 (78%)
Age in years (median, [95% CI]) (min, max)	44 [23, 70] (16, 86)	49 [31, 77] (19, 89)	44 [22, 67] (19, 74)	50 [30, 73] (23, 84)
Size in mm (median, [95% CI])	18.1 [5.6, 66.7]	28.6 [10.6, 98]	16.5 [5.7, 60.2]	28.3 [11.0, 95.2]
**A**	**A_tr_** (2005–2011)		**A_te_** (2012–2017)	
Number (% of set)	184 (22%)	646 (78%)	72 (23%)	235 (77%)
Age in years (median, [95% CI]) (min, max)	49 [25, 74] (24, 86)	56 [34, 82] (23, 89)	47 [27, 67] (27, 74)	52 [30, 74] (23, 84)
Size in mm (median, [95% CI])	12.9 [5.3, 55.8]	29.5 [8.3, 105.5]	12.7 [4.7, 55.2]	35 [9.7, 115.2]
**B**	**B_tr_** (2015–2016)		**B_te_** (2017)	
Number (% of set)	370 (26%)	1080 (74%)	111 (21%)	425 (79%)
Age in years (median, [95% CI]) (min, max)	43 [21.5, 62.5] (16, 76)	47 [30, 70] (19, 77)	43 [21, 59.2] (19, 65)	48 [30, 68] (25, 75)
Size in mm (median, [95% CI])	20.9 [5.9, 70.1]	28.2 [12.0, 90.5]	17.7 [7.2, 63.2]	27.3 [11.8, 85.5]

In Database A, the ages of 36 subjects with benign lesions and 63 subjects with cancers in the training set, and 10 subjects with benign lesions and 26 subjects with cancers in the test set, were unknown.

**Table 2:** Description of radiomic features.

**Radiomic Features Deemed Eligible for Harmonization**		
**Feature Abbreviation**	**Feature Name**	**Feature Description**
M1	Margin sharpness	Mean of the image gradient at the lesion margin
M2	Variance of margin sharpness	Variance of the image gradient at the lesion margin
M3	Variance of radial gradient histogram	Degree to which the enhancement structure extends in a radial pattern originating from the center of the lesion
T1	Contrast	Location image variations
T2	Correlation	Image linearity
T3	Difference entropy	Randomness of the difference of neighboring voxels’ gray-levels
T4	Difference variance	Variations of difference of gray-levels between voxel-pairs
T5	Energy	Image homogeneity
T6	Entropy	Randomness of the gray-levels
T7	Inverse difference moment (homogeneity)	Image homogeneity
T8	Information measure of correlation 1	Nonlinear gray-level dependence
T9	Information measure of correlation 2	Nonlinear gray-level dependence
T10	Maximum correlation coefficient	Nonlinear gray-level dependence
T11	Sum average	Overall brightness
T12	Sum entropy	Randomness of the sum of gray-levels of neighboring voxels
T13	Sum variance	Spread in the sum of the gray-levels of voxel-pairs distribution
T14	Sum of squares (variance)	Spread in the gray-level distribution
K1	Maximum enhancement	Maximum contrast enhancement
K2	Time to peak (s)	Time at which the maximum enhancement occurs
K3	Uptake rate (1/s)	Uptake speed of the contrast enhancement
K6	Enhancement at first postcontrast time point	Enhancement at first post-contrast time point
K7	Signal enhancement ratio	Ratio of initial enhancement to overall enhancement
**Radiomic Features Deemed not Eligible for Harmonization**		
**Feature Abbreviation**	**Feature Name**	**Feature Description**
S1	Volume (mm^3^)	Volume of lesion
S2	Effective diameter (mm)	Greatest dimension of a sphere with the same volume as the lesion
S3	Surface area (mm^2^)	Lesion surface area
S4	Maximum linear size (mm)	Maximum distance between any 2 voxels in the lesion
G1	Sphericity	Similarity of the lesion shape to a sphere
G2	Irregularity	Deviation of the lesion surface from the surface of a sphere
G3	Surface area/volume (1/mm)	Ratio of surface area to volume
K4	Washout rate (1/s)	Washout speed of the contrast enhancement
K5	Curve shape index	Difference between late and early enhancement
K8	Volume of most enhancing voxels (mm^3^)	Volume of the most enhancing voxels

**Table 3:** Difference in area under the receiver operating characteristic curve (ΔAUC), *p*-value for comparison, and, when ΔAUC fails to show statistically significant difference, equivalence margin for equivalence and non-inferiority, when using combinations of separate training and independent test sets. The difference in AUC (ΔAUC) is determined as AUC_post-harmonization_-AUC_pre-harmonization_. An asterisk (*) indicates statistically significant difference (including after adjusting the criteria using the Bonferroni correction for significance due to multiple comparisons, when appropriate) (A: Database A; B: Database B; tr: training set; te: test set).

Training Set	Independent Test Set	*p*-Value for ΔAUC [95% CI of ΔAUC]	Equivalence Margin (ΔAUC) for Equivalence
**Using Features Selected from Database-Specific Training Set**			
A_tr_	B_te_	<0.0001 * [0.034, 0.063]	n/a
B_tr_	A_te_	0.5 [−0.028, 0.058]	0.058
**Using Features Selected from Combined Training Set**			
(A + B)_tr_	A_te_	0.9 [−0.019, 0.017]	0.019
(A + B)_tr_	B_te_	0.17 [−0.003, 0.020]	0.020
(A + B)_tr_	(A + B)_te_	0.4 [−0.005, 0.014]	0.014

## Data Availability

No new data were created or analyzed in this study, as the radiomic features had been previously published, thus data sharing is not applicable to this article.
